# From progress to paralysis: bridging the translation gap in digital mental health care?

**DOI:** 10.1017/S0033291725102614

**Published:** 2025-12-17

**Authors:** Inez Myin-Germeys, Elisa Lievevrouw, Simona di Folco, Ine Van Hoyweghen, Luca Marelli, Michal Hajdúk, Georgia Koppe, Ulrich Reininghaus, Anita Schick, Iveta Nagyova, Jeroen Weermijer, Matthias Schwannauer

**Affiliations:** 1 Center of Contextual Psychiatry, Department of Neurosciences, KU Leuven, Leuven, Belgium; 2 Life Sciences & Society Lab, Centre for Sociological Research, KU Leuven: Katholieke Universiteit Leuven, Belgium; 3 Department of Clinical Psychology, The University of Edinburgh, UK; 4 Department of Medical Biotechnology and Translational Medicine, University of Milan, Italy; 5 Comenius University in Bratislava: Univerzita Komenskeho v Bratislave, Slovakia; 6 Department of Theoretical Neuroscience, Central Institute of Mental Health, Germany; 7 Department of Public Mental Health, Central Institute of Mental Health: Zentralinstitut fur Seelische Gesundheit, Germany; 8 Department of Social and Behavioural Medicine, Pavol Jozef Safarik University in Kosice: Univerzita Pavla Jozefa Safarika v Kos, Slovakia

Mental health problems affect over 1 billion of the global population (WHO, 2025), with a forecasted cost of over €5.5 trillion by 2030 (Bloom et al., 2011). The European Commission has therefore prioritized funding and research and innovation programs in this domain, pushing forward digital technologies as a powerful solution to make mental health care more accessible, equitable, and person-centered while providing earlier and better interventions. IMMERSE (Implementing Mobile MEntal health Recording Strategies for Europe) is such a European-funded H2020 project, examining the implementation of a novel Mobile Digital Mental Health solution (called the MoMent app) in routine mental health care across four countries in Europe (Myin-Germeys et al., 2024). While developing and testing the MoMent app, we found that the legal framework ushered in by Regulation (EU) 2017/745 on medical devices (Medical Device Regulation, MDR; European Union, 2017) hinders rather than helps the implementation of clinical and research-driven digital mental health innovations in the clinic and the wider population, despite this regulatory framework being designed to safeguard medical device effectiveness and patient safety across the continent. Based on the IMMERSE experience, we identified several barriers related to the European MDR framework, currently hampering these highly needed and desired digital innovations in mental health care.

1) *Science-based versus evidence-based apps.* According to the MDR framework, health apps are considered medical devices if manufacturers define their ‘intended use’ to be medical (e.g. monitoring, diagnostics, prevention, and treatment of disease) (Art. 2, MDR; European Union, 2017). As research-based apps, like the MoMent app in IMMERSE, undergo rigorous testing and need approval from ethical committees and competent regulatory authorities, their intended use is de facto considered ‘medical’. In contrast, many companies market their apps for mental health problems such as stress, mood, sleep, or eating behavior, as ‘lifestyle’ tools, thus avoiding the need to comply with the much stricter MDR. As a result, these companies, clinicians, hospitals, and insurance companies develop digital tools based on what they can glean from the scientific literature (labeled as *science-based)* while avoiding rigorous testing of new products in clinical studies (i.e. making them *evidence-based)*, as that would delay or even completely stop their clinical implementation. At the same time, innovative evidence-based apps with proven benefits hardly make it to the market due to the demanding MDR approval pathway.

2) *A push towards unregulated devices.* Once a digital innovation falls under MDR, it must be CE-marked before reaching the market, requiring manufacturers to comply with extensive pre- and post-market regulations (European Union, 2017). These compliance efforts are costly and burdensome. Unfortunately, research funders typically do not cover these additional costs, preventing many valuable and evidence-based clinical tools from being scaled and delivered to the public. Similarly, companies struggle to find investors for CE-marking due to the competition with lifestyle and well-being apps that do not carry these costs. This creates a distorted market where lifestyle and wellbeing devices for mental health thrive while regulated, MDR-compliant ones face financial and investment challenges.

3) *Misfit with the intrinsic character of digital tools.* The MDR broadly regulates medical devices without considering the specific needs of digital technologies. It lacks flexibility, for example, by requiring a restart of the validation process for each substantial update of the app (e.g. Annex VI 6.5.2 MDR; European Union, 2017). However, regular updates and improvements are the core and the strength of digital technologies, particularly in mental health research, where you want to flexibly adapt to the needs of clinicians and patients. In IMMERSE, we spent 5 years developing and testing the MoMent app, gathering extensive information on how to improve usability and implementation. However, including these improvements would necessitate restarting the entire MDR process. Consequently, MDR-compliant apps are often outdated.

4) *Depriving patients of helpful tools.* To establish an evidence base for innovative tools, we conduct clinical trials under the MDR framework. This means the product cannot be used outside the study unless the app is CE-marked. Due to the difficulties in financing the CE-marking of our product, we will have to withdraw the product after the trial, even if it has proved safe and effective. This deprives patients of helpful tools while exposing them to a completely unregulated market of ‘lifestyle’ apps that are not properly tested and may even be harmful. This practice seriously violates the intended patient and public protection and raises ethical concerns, as research ethics standards recommend post-trial access for participants who still need an intervention identified as beneficial in the trial (World Medical Association, n.d., par. 34).

5) *Preventing the testing of existing lifestyle tools under ‘other clinical investigation’ procedure.* Under MDR’s ‘other clinical investigation’ procedure, only commercial apps registered as medical devices from the start of production can be trialed in research and clinical studies (Art. 2, Art. 82 MDR; European Union, 2017). Apps developed for lifestyle and well-being purposes cannot gain MDR accreditation for research purposes, creating obstacles to implementing existing lifestyle apps into intervention and clinical practice. Despite their widespread use by the public to support mental health and well-being, their effectiveness cannot be clinically proven. It is, thus, impossible to create an evidence base for many existing (mental) health-related digital tools, excluding them from clinical use and best practice guidelines. While medical devices and lifestyle apps are treated as separate categories in the regulatory framework, clinicians and the public often use both as they access simply what is available, rather than what is evidence-based.

The current legislative and regulatory framework impedes progressive innovation in digital health in clinical research and widens the gap between community-based prevention and evidence-based medicine. We require a broader and more adaptable regulatory framework that supports the safe development of digital interventions and tools in research and clinical contexts. This framework should allow clinical research to catch up with commercial developments and establish a robust evidence base for existing tools. Regulation of digital health innovations and non-invasive app-based tools needs to evolve to align with their development cycle and be in sync with clinical and applied research. Public services must have pathways for innovation and quality improvement in clinical care that embrace digital advancements and ensure that tools used in clinical and preventive settings are as accessible to a critical, developing evidence base as other medical innovations. It would require an internationally recognized multi-stakeholder body, akin to the National Institute for Clinical Excellence (NICE; National Institute for Health and Care Excellence, 2025), to develop best practice guidelines for the governance and development of digital health and mental health tools, providing a framework for the revision of legal and ethical regulations in this rapidly evolving field.
